# Retrospective Evaluation of Cryoprecipitate Transfusion in Dogs to Prevent or Treat Hemorrhage: 21 Cases (2009–2023)

**DOI:** 10.1111/vec.70045

**Published:** 2025-10-07

**Authors:** W. Y. Eunice Lam, Linda G. Martin, K. Jane Wardrop, Jillian M. Haines

**Affiliations:** ^1^ Department of Veterinary Clinical Sciences, College of Veterinary Medicine Washington State University Pullman Washington USA

**Keywords:** cryoprecipitate, von Willebrand disease, von Willebrand factor

## Abstract

**Objective:**

To report homologous cryoprecipitate transfusions in dogs with hemostatic disorders, hemorrhage, or risk of hemorrhage, and to report adverse reactions associated with these cryoprecipitate transfusions.

**Design:**

Retrospective case series (July 2009 to July 2023).

**Setting:**

University teaching hospital.

**Animals:**

Twenty‐one client‐owned dogs with hemostatic disorders, hemorrhage, or risk of procedure‐related hemorrhage.

**Interventions:**

None.

**Measurements and Main Results:**

All dogs received homologous cryoprecipitate transfusion.

Sixteen dogs had von Willebrand disease (one also had factor XI deficiency); three dogs had hemophilia A; and two dogs had normal concentration of von Willebrand factor and were not diagnosed with any hemostatic disorder. Sixteen dogs also received other blood products and/or hemostatic medications. Twenty‐two cryoprecipitate transfusions were administered to 16 dogs before, during, or after surgery; 16 of those 22 transfusions were given to 14 dogs without evidence of hemorrhage but with history of bleeding disorder and/or previous hemorrhage with the aim of preventing hemorrhage. Eight transfusions were given to six dogs to control hemorrhage not associated with surgery. Sixteen dogs (18 transfusions) received cryoprecipitate prepared in‐house. Five dogs (12 transfusions) received a commercial lyophilized cryoprecipitate. No dog developed any serious adverse reactions to cryoprecipitate transfusion. All dogs were discharged from the hospital (median, 2 days after cryoprecipitate transfusion [range, 1–25 days]). The benefits of cryoprecipitate transfusion could not be well documented because of the retrospective nature of this study and the concurrent administration of other blood products and/or hemostatic medications to 16 dogs.

**Conclusions:**

Homologous cryoprecipitate transfusion appeared to be safe when administered for prevention or treatment of hemorrhage in dogs, but additional studies to assess safety and efficacy are warranted.

**Abbreviations**
aPTT: activated partial thromboplastin time
BMBT: buccal mucosal bleeding time
CI: confidence interval
cryo: cryoprecipitate
FFP: fresh frozen plasma
FNHTR: febrile non‐hemolytic transfusion reaction
HA: hemophilia A
PT: prothrombin time
TRACS: Transfusion Reaction Small Animal Consensus Statement
vWD: von Willebrand disease
vWf: von Willebrand factor
YB: Yunnan Baiyao

## 1 Introduction

Cryoprecipitate (cryo), the insoluble fraction of fresh frozen plasma (FFP) after thawing at 1°C–6°C (33.8°F–42.8°F), is rich in coagulation factor VIII, fibrinogen, and von Willebrand factor (vWf) [1, 2]. A bottle of lyophilized canine cryo^1^ (about 70 mL after reconstitution with 0.9% NaCl) contains at least 80 IU of factor VIII, 150 mg of fibrinogen, and 80 IU of vWf, with these constituents being equivalent to 250–300 mL of canine FFP [3].

Homologous cryo transfusion is currently recommended for dogs with von Willebrand disease (vWD) and hemophilia A (HA) that have significant bleeding [4]. Cryo transfusion may also be used prophylactically (e.g., before surgery or biopsy) when dogs are at high risk of significant bleeding [4, 5]. The only contraindication for cryo transfusion mentioned by the manufacturer of the commercial lyophilized canine cryo^a^ is previous anaphylactic reaction to any plasma containing products [3].

The risk of complications associated with cryo transfusion in dogs has not been investigated. Transfusion of homologous blood products may cause adverse reactions such as fever, tachycardia, changes in respiratory pattern, allergic reactions, nausea, vomiting, diarrhea, hemolysis, anemia, hypocalcemia, hyperammonemia, hypotension, and purpura [6]. Anaphylactic reactions from canine cryo are thought to be extremely rare [3, 4]. As of October 2023, only three publications reporting a total of 19 dogs treated with homologous cryo transfusion could be found [5, 7, 8]. In the only reported case of transfusion reactions in a dog receiving cryo, gastrointestinal signs (vomiting and diarrhea) and purpura were observed after multiple cryo transfusions given during three hospital visits, over a 2‐year period. Fortunately, the dog responded to treatment and survived to discharge [7].

The objectives of this descriptive study were to report the use of homologous cryo transfusion for prevention and treatment of hemorrhage in dogs, and to assess the occurrence of transfusion reactions.

## 2 Materials and Methods

### 2.1 Preparation and Administration of Cryoprecipitate

Either cryo prepared in‐house at Washington State University Veterinary Teaching Hospital or a lyophilized commercial product^a^ was used in all cases. The commercial lyophilized cryo^a^ was used as recommended by the manufacturer [3]. In‐house preparation of cryo was performed as described in Box 1. Cryo transfusions were administered IV at a rate of 2–5 mL/kg/h with a syringe pump and a standard blood administration set attached to the IV catheter. Then, 0.9% NaCl was used to flush the remaining cryo through the extension set to complete the transfusion.

**BOX 1: In‐house preparation of cryoprecipitate from canine fresh frozen plasma**

1. Canine fresh frozen plasma is thawed in a refrigerator at 1°C–6°C for 5–15 h until only a small amount of ice crystals remained;
2. The bag is centrifuged (4000 RPM, 8 min, 4°C) and gently removed from the centrifuge;
3. Approximately 3 mL of cryoprecipitate remains in the bottom of the bag or is “coating” the sides of the bag;
4. The bag is inverted to allow supernatant plasma to flow by gravity into the satellite bag;
5. A total of 10–15 mL of plasma is left in the satellite bag;
6. The cryoprecipitate bag is labeled and immediately frozen.

### 2.2 Monitoring for Signs of Adverse Transfusion Reactions

Heart rate, respiratory rate, temperature, blood pressure, and signs of potential adverse transfusion reactions were assessed prior to transfusion and 5 min, 10 min, 15 min, 1 h, 2 h, 3 h, and 4 h after the start of the transfusion.

### 2.3 Data Collection

Using the keywords “canine,” “transfusion,” and “cryoprecipitate,” the medical records of dogs treated at the Washington State University Veterinary Teaching Hospital with homologous cryo between July 1, 2007, and July 31, 2023, with the aim of preventing or treating hemorrhage were reviewed. Cases of cryo transfusion without documentation of hemorrhage or increased risk of hemorrhage were excluded from the study. Data recorded included signalment (age, breed, sex); body weight; history; clinical signs; diagnostic tests and treatment before admission; presence of cardiac disease; volume of cryo administered; justification for cryo transfusion; product used and circumstances of cryo administration (e.g., before, during, or after surgery, or to manage bleeding not associated with surgery); other medications that could affect hemostasis (e.g., other blood products, hemostatic medications, nonsteroidal anti‐inflammatory drugs) used prior, during, or after cryo transfusion; hemostasis analyses (e.g., buccal mucosal bleeding time [BMBT], prothrombin time [PT], activated partial thromboplastin time [aPTT], concentration of vWf) prior to and after cryo transfusion; possible or confirmed adverse transfusion reactions to cryo administration; short‐term outcome (survival to discharge); and treatment cost.

### 2.4 Transfusion Reaction Definitions

The Transfusion Reaction Small Animal Consensus Statement (TRACS) was used to classify any potential transfusion reaction in the present retrospective study [6]. Imputability (cause) of any potential transfusion reaction was classified based on the Hemovigilance Module developed by the Centers for Disease Control and Prevention and recently adapted to veterinary medicine [9]. Febrile non‐hemolytic transfusion reaction (FNHTR) was defined as temperature >39.2°C (102.5°F) during or within 4 h of the end of a transfusion and an increase in temperature of >1°C (1.8°F) from the pretransfusion body temperature. Transfusion‐associated dyspnea was defined as development of acute respiratory distress during or within 24 h of the end of a transfusion. Hypotensive transfusion reaction was defined as rapid onset of hypotension during or shortly after the transfusion without other explanation [6]. Evidence of any possible transfusion reaction was reviewed a second time by the authors of this case series to reach a consensus on the type, imputability, and severity of each transfusion reaction.

### 2.5 Data Analysis

Descriptive data analysis was conducted. The cases were grouped by cryo product (i.e., in‐house vs. commercial), bleeding disorder (e.g., vWD vs. other conditions), aim of treatment (e.g., prophylaxis vs. treatment of hemorrhage), and whether cryo transfusion was administered to prevent/treat hemorrhage associated with surgery or not. Comparisons between groups were performed with the Mann–Whitney test (for continuous variables) or the Fisher's exact test (for proportions). The Fisher's exact test was also used to compare the incidence of adverse reactions in the present case series to the pooled incidence of transfusion reactions from all published case series including at least 100 dogs transfused with plasma or any plasma product. The level of significance was set at α = 0.05 for all comparisons. The confidence intervals (CIs) for the incidence of transfusion reactions were also calculated.

## 3 Results

### 3.1 Study Population

Twenty‐two cases of cryo transfusion in dogs were found in the medical records search. One case that did not meet the inclusion criteria was removed from the study: a dog with advanced neoplasia, gastric perforation, and sepsis that neither had hemorrhage nor was subjected to any procedure (surgery, biopsy or dental extraction) that could have caused hemorrhage. Twenty‐one dogs received homologous cryo for prevention or treatment of hemorrhage on 30 occasions during the study period. Doberman Pinscher or a Doberman cross was the most common breed (*n* = 9 [42.9%]), followed by American Eskimo Dog (*n* = 3 [14.3%]), Border Collie or Border Collie cross (*n* = 2 [9.5%]), and Great Dane, Yorkshire Terrier, German Shepherd Dog, Australian Cattle Dog, German Shorthaired Pointer, and Field Spaniel (*n* = 1 each [4.8%]). Nine were females (42.9%) (eight intact), and 12 were males (57.1%) (eight intact). The median age was 2.5 years (range, 0.1–11.0 years). The median body weight was 26.4 kg (range, 2.0–54.3 kg). Prior to cryo transfusion, 16 dogs (76.2%) were diagnosed with vWD (one of them also had factor XI deficiency); three dogs (14.3%) were diagnosed with HA; two dogs (9.5%) (a Doberman Pinscher and a German Shorthaired Pointer) were not diagnosed with a primary hemostatic disorder, and their vWf concentration was within normal limits; and two (9.5%) Doberman Pinschers diagnosed with vWD also had cardiac disease (one had degenerative valvular disease, subaortic stenosis, mild mitral regurgitation likely due to endocardiosis, trivial tricuspid regurgitation, and pulmonic valve insufficiency; the other dog had subaortic stenosis, diastolic dysfunction, and mitral valve regurgitation likely due to endocardiosis) but were not receiving any medication for their cardiac conditions.

### 3.2 Cryoprecipitate Transfusion

Twenty‐two (73.3%) of the 30 treatments (median cryo dose, 5.0 mL/kg [range, 0.9–18.5]) were administered to 16 dogs before, during, or after surgery. Sixteen treatments (53.3%) were given before surgery to 14 dogs without any evidence of hemorrhage because those 14 dogs had known bleeding disorders or history of previous hemorrhage (Table S1). Eight (26.7%) of the 30 treatments (median cryo dose, 8.1 mL/kg [range, 6.8–12.5]) were given to six dogs with the aim of controlling hemorrhage not associated with surgery (Table S2).

Cryo prepared in‐house was administered to 16 dogs (76.2%) on 18 occasions (60.0%) (two dogs [dogs 6 and 10] were treated twice). A commercial lyophilized cryo^a^ was administered to five dogs (23.8%) on 12 occasions (40.0%) (one dog received one transfusion per visit during four visits, and four additional cryo transfusions [one transfusion per day] during one visit).

Before admission, the referring veterinarian treated one dog with whole blood and vitamin K_1_ 2 because of hemorrhage, anemia, and a presumptive diagnosis of rodenticide toxicosis (Table S1). After admission, five dogs undergoing surgery (Table S1) and two dogs not requiring surgery (Table S2) received blood products other than cryo (FFP, whole blood, packed red blood cells^3^, platelet‐rich plasma, or platelet concentrate). Six dogs undergoing surgery (Table S1) and three dogs not requiring surgery (Table S2) received a hemostatic medication other than a blood product. Four dogs were treated with tranexamic acid^4^, four dogs with Yunnan Baiyao (YB)^5^, and two dogs with desmopressin acetate^6^. Three dogs were medicated with carprofen^7^ after surgery (one before admission to our hospital).

Before cryo administration, six dogs (28.6%) had BMBT measured, and in all six dogs, BMBT was longer than 5 min. Only two of those dogs had a follow‐up BMBT, 6 and 11 days after the cryo transfusion, respectively, when the BMBT was normal. Justification for the BMBT retest so many days after cryo transfusion for these dogs could not be found in the medical records. Of the eight dogs that had PT measured before cryo administration, one dog had prolonged PT. Unfortunately, PT was not measured again after cryo transfusion. Of the nine dogs that had aPTT measured before cryo administration, two had a prolonged aPTT. In one of those dogs, the aPTT was still elevated 48 h after cryo administration but returned to the normal range 11 days after treatment. In this dog, which had been admitted with post‐castration hemorrhage, D‐dimer concentration in plasma was markedly elevated (>2000 ng/mL) 2 days after cryo transfusion. In one dog that did not have an aPTT measured before cryo administration, the aPTT was prolonged 24 h after treatment.

### 3.3 Transfusion Reactions

Due to inconsistent recording of the occurrence of adverse transfusion reactions and vital signs during and after cryo administration, five dogs (23.8%) that received cryo on seven occasions (23.3%) (one dog received cryo on three occasions) were excluded from the assessment of the incidence of adverse transfusion reactions to cryo. Therefore, only 16 dogs (76.2%) that received a cryo transfusion on 23 occasions (76.7%) were included in the calculation of the incidence of adverse reactions to cryo. Only three of those dogs (18.8% [95% CI: 4.1%–45.7%]) had clinical signs suggestive of transfusion reactions to cryo administration on three occasions (one transfusion per dog) (13.0% [95% CI: 2.8%–33.6%]) (Tables S1 and S2). The incidence of transfusion reactions to cryo in the present case series was not different (*p* = 0.41) from the pooled incidence of transfusion reactions of 7.6% (95% CI: 6.0%–9.4%) observed in three previous reports in dogs including 975 plasma product transfusions [8, 10, 11].

All three dogs with clinical signs suggestive of transfusion reaction received other blood products in addition to cryo, but not at the same time as the cryo transfusion. Two of those dogs received cryo prepared in‐house. One dog became restless 15 min after the start of the cryo transfusion. Another dog, which had normal temperature 7 h prior to the cryo transfusion, had a mildly elevated rectal temperature (39.4°C [103°F]) 1 h after starting cryo administration. A third dog was panting 30 min after initiation of the cryo transfusion and had a decline in systolic blood pressure (90 mm Hg) for approximately 30 min starting at the end of the cryo transfusion. Based on the TRACS guidelines for assessment and classification of transfusion reactions in dogs and cats [6] and the hemovigilance program at a veterinary teaching hospital [9], the transfusion reactions observed in these three dogs were classified as doubtful transfusion‐associated dyspnea, possible FNHTR, and doubtful hypotensive transfusion reaction or transfusion‐associated dyspnea, respectively. In all three cases, the transfusion reactions were classified as nonsevere. In two of the dogs, the respiratory signs and fever resolved without any medical intervention within 15 min and 2 h during cryo transfusion, respectively. In the third dog, the systolic blood pressure normalized (102 mm Hg) after administration of an IV bolus of isotonic fluid after cryo administration, and recovery was uneventful.

Two other dogs that underwent surgery during three visits (spinal surgery and mass removal from rectum in one dog, unilateral mastectomy in another) also developed mild hypotension while under general anesthesia and shortly after cryo transfusion. These two dogs did well after anesthesia recovery, and the hypotension was not considered as an adverse transfusion reaction. Nine additional dogs (eight had surgery; one underwent magnetic resonance imaging) also received the cryo treatment during general anesthesia, but hypotension was not recorded in these cases.

### 3.4 Final Outcome

All dogs were discharged from the hospital (median, 2 days after cryo transfusion [range, 1–25 days]) (median duration of hospitalization, 3 days [range, 1–29 days]) (Tables S1 and S2). Duration of hospitalization was not affected by the cryo product used, hemostatic disorder, or justification of cryo transfusion (e.g., prophylaxis or treatment of hemorrhage) but was longer (*p* = 0.019) in dogs that underwent surgery (median, 3.5 days [range, 1–29 days]) (Table S1) when compared to the dogs not subjected to surgery (median, 2 days [range, 1–3 days]) (Table S2). Although the cost of the in‐house cryo was lower than the commercial product (*p* = 0.010), the type of cryo used did not affect the total bill (*p* = 0.418). The cryo product used, hemostatic disorder, prophylactic or therapeutic use of cryo, and whether the dogs were subjected to surgery were not associated with any other outcome variable including the incidence of complications.

## 4 Discussion

This study describes the use of cryo transfusion for prevention or treatment of hemorrhage in dogs. Since all dogs survived to discharge and no dog developed any signs of serious adverse transfusion reaction to the cryo, the use of this treatment was likely safe. Furthermore, neither the cryo prepared in‐house nor the commercial product was cost prohibitive, which further supports the use of cryo transfusion in dogs. However, the benefits of cryo transfusion could not be well documented in this study because there was no control group (i.e., dogs with similar clinical conditions not treated with cryo), seven dogs had also received other blood products, and 11 dogs had received other hemostatic medications.

Using the TRACS guidelines for assessment and classification of transfusion reactions in dogs and cats [6] and the recently published hemovigilance program at veterinary teaching hospitals to classify the reactions based on severity and imputability (cause) [9], and taking a conservative approach, the incidence of transfusion reactions to cryo in the present study was relatively high (18.8% of the dogs; 13.0% of the transfusions). This finding contrasts with the absence of transfusion reactions in two recent case series of cryo transfusion in dogs including four transfusions combined with desmopressin [5] and 14 transfusions without the administration of any hemostatic medication [8]. The relatively high incidence of transfusion reactions in dogs receiving cryo in the present study, which was not different than the pooled incidence of transfusion reactions observed in three previous reports in dogs including 975 plasma product transfusions [8, 10, 11], was not expected considering that fewer proteins are administered when cryo is transfused relative to plasma transfusion [12, 13]. On a positive note, the three instances of transfusion reaction observed in the present study were classified as nonsevere; two of those instances were considered doubtful and the other instance possible; two of the dogs recovered within 2 h without any intervention, and the third dog recovered uneventfully after fluid therapy.

The mild hypotension shortly after cryo transfusion observed on three occasions in two dogs during surgery under general anesthesia was likely secondary to general anesthesia and not a transfusion reaction. Hypotension is highly prevalent during general anesthesia [14, 15, 16], and those two dogs did well after anesthesia recovery. For that reason, those instances of hypotension were not classified as transfusion reactions.

Since only 30 cryo transfusions were administered to 21 dogs included in the present case series, the apparent safety of cryo administration should not be overinterpreted. The relatively small number of cases during the study period certainly minimized the odds of encountering adverse effects, especially the less common transfusion reactions [6]. Furthermore, the fact that, on multiple occasions, cryo was given when the dog was under general anesthesia could have compromised the clinicians’ ability to detect signs of adverse transfusion reactions. General anesthesia affects some of the main variables (e.g., heart rate, respiratory rate, temperature, blood pressure) [14, 15, 16] used to detect adverse transfusion reactions [6].

Only one case of a transfusion reaction to canine cryo has been previously reported: A dog with HA, anemia, and swelling of a pelvic limb received multiple transfusions of cryo prepared in‐house on two hospital admissions, 2 years apart. Both times, the dog developed multiple adverse reactions after cryo transfusion such as diarrhea, vomiting, and purpura (swelling, thrombocytopenia, petechia, and ecchymoses). The transfusion reactions were managed with oral prednisone, FFP, and platelet‐rich plasma [7]. Fortunately, such severe transfusion reactions were not observed in the present study.

Von Willebrand disease, the most prevalent hemostatic disorder in the present study, is known to be the most common inherited hemostatic disorder in dogs [17]. Doberman Pinschers, the most common breed diagnosed with vWD in the present study, are known to have a high prevalence of vWD [18]. Although three male American Eskimo Dogs were the only dogs diagnosed with HA in the present study, the fact that the three were siblings should explain this apparent breed predisposition. Indeed, no evidence that this breed is predisposed to HA has been reported. HA, a deficiency of factor VIII, is expressed almost exclusively in male dogs [19, 20, 21] due to its x‐linked recessive inheritance [17, 22], and mixed breed dogs, German Shepherd Dogs, and Labrador Retrievers are the most commonly reported breeds [20, 22].

Deficiencies of specific coagulation factors such as vWf and factor VIII (which together accounted for 90% of dogs in this study) can be managed with plasma transfusions [5, 20]. However, considering the relatively low concentration of vWf and factor VIII in plasma [1], the FFP approach would require a larger volume transfusion and would provide excessive amounts of other components such as water, electrolytes, and albumin. Cryo transfusion is a better approach for replacement of specifically vWf and factor VIII [17, 23] as it helps avoid volume overload that can be caused by plasma transfusion, which is particularly important in dogs with concurrent cardiac disease, such as two of the dogs in the present series.

In the present study, blood products other than cryo and the hemostatic medications Vitamin K_1_, tranexamic acid, YB, and desmopressin were administered to several dogs prior, during, or after cryo transfusion (Tables S1 and S2). The administration of blood products other than cryo and the administration of hemostatic medications may have contributed to improved hemostasis and minimized hemorrhage, which was also the goal of the cryo transfusions. Thus, these other treatments prevented the full assessment of the benefits of the cryo transfusions.

In the present study, blood products other than cryo were administered to seven dogs (FFP to four, packed red blood cells to three, whole blood to two, and platelet‐rich plasma and platelet concentrate to one each). None of these other blood products is comparable to cryo as a source of vWf or factor VIII [1], the blood components most in need for the control or prevention of hemorrhage in the group of 21 dogs of the present study, 19 of which were diagnosed with vWD or HA. However, the other blood products administered to most of the dogs in the present study provided other essential components such as red blood cells, platelets, and albumin and may have helped to improve the patients’ conditions.

The lipophilic vitamin K_1_, which is essential to reactivate the procoagulant factors II, VII, IX, and X [24], was administered prior to admission to one dog in the present study because its attending veterinarian suspected coumarin‐based anticoagulant toxicosis. Treatment with vitamin K_1_ combined with various blood products (e.g., FFP, stored plasma, or whole blood) is the standard treatment for dogs with acute hemorrhage associated with ingestion of a coumarin‐based anticoagulant [25].

The synthetic derivate of lysine with antifibrinolytic properties, tranexamic acid, was used in four surgical cases for hemorrhage prophylaxis or control in the present study. A previous study in 11 healthy dogs showed safety and effectiveness of IV administration of tranexamic acid, which improved PT [26]. Tranexamic acid administration in people undergoing elective surgery resulted in a reduction in blood transfusion requirement by a third compared to people not treated with tranexamic acid [27]. Also, in people, co‐administration of tranexamic acid and cryo transfusion minimizes mortality associated with combat injuries relative to exclusive administration of cryo [28].

The Chinese herbal supplement Yunnan Baiyao, a medication not well tested in dogs, has been used extensively in dogs with hemorrhage [29]. YB was administered orally to four dogs in the present study. In one study conducted in dogs [30], thromboelastography revealed that YB administered orally increased the strength of the clot in healthy dogs. Only one of the 18 dogs had a possible adverse reaction, mild self‐limiting diarrhea [30]. And experimental study found that YB shortened bleeding time in rats and reduced in vitro blood clotting time of rabbit blood [31]. In a few studies in people, YB reduced bleeding associated with surgery and ulcerative conditions [32, 33, 34].

The synthetic derivate of vasopressin, desmopressin, which was used in two dogs in this series, has been used to treat hemorrhage associated with canine HA, vWD, and factor VII deficiency [5, 35, 36]. Intravenous administration of desmopressin has also been reported in three dogs with acetylsalicylic acid‐induced bleeding. Desmopressin reversed the markedly prolonged BMBT and minimized intraoperative bleeding [37]. In one recent case series of laparoscopic ovariohysterectomy or ovariectomy in 16 dogs with vWD and four dogs with factor VII deficiency, all 16 dogs with vWD received desmopressin and four of those dogs also received a cryo transfusion prior to surgery; one of the four dogs with factor VII deficiency received desmopressin. No postoperative complications, including hemorrhage, were reported in those 17 dogs treated with desmopressin [5].

Carprofen, a nonsteroidal anti‐inflammatory drug that could negatively affect hemostasis, was administered to three dogs in the present study. In a previous study, 10 healthy dogs treated with carprofen had decreased platelet aggregation and prolonged aPTT [38]. In the present study, carprofen did not obviously compromise the antihemorrhagic effect of cryo transfusion.

The present study highlighted the apparent safety of homologous cryo transfusion for prevention or treatment of hemorrhage in dogs with hemostatic disorders. However, this study had the limitations of its retrospective approach, including inconsistencies in the medical records, the lack of a control group, and the relatively small number of cases. The low frequency of cryo transfusions in dogs at our institution (average, 1.3 cryo transfusions per year) justified the retrospective approach of the present study [39]. To effectively assess the pros and cons of cryo transfusion in dogs with hemostatic disorders, a large multicenter, prospective, randomized case–control study could be considered [40]. However, this would not be feasible without withholding appropriate medical care to patients.

It can be concluded that the administration of homologous cryo transfusion for prevention or treatment of hemorrhage in dogs appeared to be relatively safe. However, conclusions about the efficacy of canine cryo cannot be drawn without additional investigation.

## Author Contributions

**W. Y. Eunice Lam**: conceptualization, data curation, formal analysis, investigation, methodology, project administration, resources, software, validation, visualization, writing – original draft, writing – review and editing. **Linda G. Martin**: conceptualization, supervision, validation, writing – review and editing. **K. Jane Wardrop**: conceptualization, supervision, validation, writing – review and editing. **Jillian M. Haines**: conceptualization, supervision, validation, writing – review and editing.

## Conflicts of Interest

The authors declare no conflicts of interest.

## Supporting information




**Supplemental Table 1**: – Sixteen dogs treated with homologous cryoprecipitate for prophylaxis or control of hemorrhage associated with surgery—History, clinical findings, hemostatic disorder, other blood products administered, hemostatic medication administered and discharge day.

**Supplemental Table 2**: – Six dogs treated with homologous cryoprecipitate for control of hemorrhage not associated with surgery—History, clinical findings, hemostatic disorder, other blood products administered, hemostatic medication administered and discharge day.

## Endnotes

1 Canine Cryoprecipitate Lyophilized, ABRINT, Stockbridge, MI.

2 Vitamin K, Sparhawk Laboratories, Lenexa, KS.

3 Canine Packed Red Blood Cells, Hemopet, Garden Crove, CA.

4 Tranexamic Acid Injection, USP, Indoco Remedies Limited, Verna, Goa, India.

5 Yunnan Baiyao Capsules, Yunnan Baiyao Group Corp. Ltd, Kumming, China.

6 Desmopressin Acetate Injection, USP, Sun Pharmaceutical Industries, Inc., Cranbury, NJ.

7 Carprofen, Zoetis, Kalamazoo, MI.
