# Supplementary material for: Retrospective Evaluation of Cryoprecipitate Transfusion in Dogs to Prevent or Treat Hemorrhage: 21 Cases (2009–2023)
Source: J Vet Emerg Crit Care (San Antonio). 2025 Oct 7;35(5):564–70. doi: 10.1111/vec.70045 (PMC12614411; doi:10.1111/vec.70045)
Supplement: Supplementary file 1 — Supplemental Table 1: – Sixteen dogs treated with homologous cryoprecipitate for prophylaxis or control of hemorrhage associated with surgery—History, clinical findings, hemostatic disorder, other blood products administered, hemostatic medication administered and discharge day. [file VEC-35-564-s002.docx]

Supplemental Table 1 – Sixteen dogs treated with homologous cryoprecipitate for prophylaxis or control of hemorrhage associated with surgery – History, clinical findings, hemostatic disorder, other blood products administered, hemostatic medication administered and discharge day.

| **Dog** | **History and clinical findings** | **Hemostatic disorder** | **Surgery** | **Treatment goal** | **Cryo dose (mL/kg)** | **Other blood products** | **Hemostatic medication** | **Discharge day post admission (post cryo)** |
| --- | --- | --- | --- | --- | --- | --- | --- | --- |
| 1 | hematuria at 2 months | vWD | ovariohisterectomy | prophylaxis | 2.9 | FFP | DDAVP, TA | 3 (3) |
| 2 | 5-day wound hemorrhage after castration, thrombocytopenia | vWD | castration | control | not recorded | FFP, WB, PRP | - | 14 (11) |
| 3 | no clinical signs | vWD | laparoscopic ovariectomy & dental extractions | prophylaxis | 5.7 | - | - | 1 (1) |
| 4 | progressive tetraparesis & spinal pain | vWD | spinal surgery | prophylaxis | not recorded | - | - | 29 (25) |
| 5 | hematoma post venipuncture | vWD | laparoscopic ovariectomy & dental extractions | prophylaxis | 18.5 | - | - | 3 (2) |
| 6 | tetraparesis & ataxia | vWD | spinal surgery | prophylaxis | not recorded | - | - | 29 (24) |
| 6 | bloody stool & rectal mass | vWD | rectal mass resection | prophylaxis | 0.9 | - | - | 2 (1) |
| 7 | no clinical signs | vWD | castration | prophylaxis | 2.0 | - | - | 3 (2) |
| 8 🞊 | anemia, lethargy, bleeding ears, fever, presumptive rodenticide toxicosis | vWD | bone marrow aspirate & biopsy | prophylaxis | 4.9 | WB | Vitamin K_1_ * | 5 (5) |
| 10 | hematoma after spinal surgery | none | hematoma debridement | prophylaxis | 2.8 | FFP | - | 13 (10) |
| 10 | 2nd treatment given during surgery | none | hematoma debridement | control | not recorded | - | - | 13 (9) |
| 11 | no clinical signs | vWD | laparoscopic cryptorchidectomy | prophylaxis | 1.6 | - | - | 2 (1) |
| 12 | self-mutilated tail | vWD | laparoscopic ovariectomy &  partial tail amputation | prophylaxis | 1.0 | - | TA | 3 (2) |
| 13 # | no clinical signs | vWD | laparoscopic ovariectomy | prophylaxis | 4.6 | - | DDAVP | 2 (1) |
| 14 | hemorrhage at & around surgical incision & at venipuncture sites | none | mastectomy | control | 5.0 | - | TA | 2 (2) |
| 16 # | severe periodontal disease & bleeding | HA | dental extractions | prophylaxis | 7.4 | - | YB | 1 (1) |
| 16 # | no clinical signs | HA | dental extractions | prophylaxis | 8.5 | - | TA | 6 (5) |
| 16 # | mild-moderate post-op. hemorrhage | HA | dental extractions | control | not recorded | pRBC | YB | 6 (4) |
| 16 # ⊗ | mild-moderate post-op. hemorrhage | HA | dental extractions | control | 8.5 | pRBC | YB | 6 (3) |
| 16 # | mild-moderate post-op. hemorrhage | HA | dental extractions | control | 8.5 | pRBC | YB | 6 (2) |
| 18 # | no clinical signs | vWD | ovariohisterectomy | prophylaxis | 6.6 | - | - | 4 (4) |
| 20 | mammary masses, no bleeding | vWD & factor XI deficiency | mastectomy | prophylaxis | 5.3 | - | - | 1 (1) |
| **Median** |  |  |  |  | 5.0 |  |  | 3.5 (2.5) |
| **Min** |  |  |  |  | 0.9 |  |  | 1 (1) |
| **Max** |  |  |  |  | 18.5 |  |  | 29 (25) |

Each row represents one cryo transfusion administered to a different patient or on a different day; 🞊 Dog with possible nonsevere transfusion reaction observed during or after the administration of cryoprecipitate; ⊗ Dog with doubtful nonsevere transfusion reaction observed during or after the administration of cryoprecipitate; # Dog treated with commercial cryoprecipitate (i.e., the other dogs were treated with in-house cryoprecipitate); HA=Hemophilia A; * Medication administered prior to hospital admission; none=no hemostatic disease diagnosed and vWD ruled out (i.e., normal concentration of von Willebrand factor); FFP=Fresh frozen plasma; pRBC=Packed red blood cells; PC=Platelet concentrate; PRP=Platelet rich plasma; WB=Whole blood; DDAVP=Desmopressin acetate; TA=Tranexamic acid; YB=Yunnan Baiyao
