# Supplementary material for: Retrospective Evaluation of Cryoprecipitate Transfusion in Dogs to Prevent or Treat Hemorrhage: 21 Cases (2009–2023)
Source: J Vet Emerg Crit Care (San Antonio). 2025 Oct 7;35(5):564–70. doi: 10.1111/vec.70045 (PMC12614411; doi:10.1111/vec.70045)
Supplement: Supplementary file 2 — Supplemental Table 2: – Six dogs treated with homologous cryoprecipitate for control of hemorrhage not associated with surgery—History, clinical findings, hemostatic disorder, other blood products administered, hemostatic medication administered and discharge day. [file VEC-35-564-s001.docx]

Supplemental Table 2 – Six dogs treated with homologous cryoprecipitate for control of hemorrhage not associated with surgery – History, clinical findings, hemostatic disorder, other blood products administered, hemostatic medication administered and discharge day.

| **Dog** | **History and clinical findings** | **Hemostatic disorder** | | **Cryo dose (mL/kg)** | **Other blood products** | **Hemostatic medication** | **Discharge day post admission (post cryo)** |
| --- | --- | --- | --- | --- | --- | --- | --- |
| 9 ⊗ | bleeding gingiva, melena & vomited blood, 5 bleeding events in the last 4 years | vWD | | not recorded | pRBC, FFP | - | 1 (1) |
| 15 # | stiff gait, previous bleeding (toe & gastrointestinal), MRI under GA, hematomyelia | HA | | 12.5 | - | YB | 3 (2) |
| 16 # | severe hematoma of right hindlimb | HA | | 11.5 | - | - | 2 (2) |
| 16 # | bleeding at bite wound | HA | | 6.9 | - | - | 2 (2) |
| 16 # | acute oral bleeding, coughing blood, melena, low PCV | HA | | 6.8 | - | - | 2 (2) |
| 17 # | active bleeding at paw laceration despite bandaging | HA | | 8.5 | - | YB | 3 (3) |
| 19 | severe anemia & active bleeding from gingiva | vWD | | not recorded | PC, pRBC | YB | 1 (1) |
| 21 | prolonged (>8 hours), mild bleeding from right maxillary gum laceration | vWD | | 7.6 | - | - | 1 (1) |
| **Median** |  | |  | 8.1 |  |  | 2 (2) |
| **Min** |  | |  | 6.8 |  |  | 1 (1) |
| **Max** |  | |  | 12.5 |  |  | 3 (3) |

Each row represents one cryoprecipitate transfusion administered to a different patient or on a different day; ⊗ Dog with probable nonsevere transfusion reaction observed during or after the administration of cryoprecipitate; # Dog treated with commercial cryoprecipitate (i.e., the other dogs were treated with in-house cryoprecipitate); HA=Hemophilia A; pRBC=Packed red blood cells; FFP=Fresh frozen plasma; PC=Platelet concentrate; YB=Yunnan Baiyao
